# Configuration and Optimization of a Minichannel Using Water–Alumina Nanofluid by Non-Dominated Sorting Genetic Algorithm and Response Surface Method

**DOI:** 10.3390/nano10050901

**Published:** 2020-05-08

**Authors:** Ali Akbar Ahmadi, Masoud Arabbeiki, Hafiz Muhammad Ali, Marjan Goodarzi, Mohammad Reza Safaei

**Affiliations:** 1 Department of Mechanical Engineering, Isfahan University of Technology, Isfahan 84156-83111, Iran; ali.akb.ahmadi@gmail.com; 2 Department of Mechanical Engineering, Payame Noor University, Tehran 19395-3697, Iran; m.arabbeiki@yahoo.com; 3 Mechanical Engineering Department, King Fahd University of Petroleum and Minerals, Dhahran 31261, Saudi Arabia; hafiz.ali@kfupm.edu.sa; 4 Sustainable Management of Natural Resources and Environment Research Group, Faculty of Environment and Labour Safety, Ton Duc Thang University, Ho Chi Minh City 700000, Vietnam; marjan.goodarzi@tdtu.edu.vn; 5 Institute of Research and Development, Duy Tan University, Da Nang 550000, Vietnam; 6 Faculty of Electrical—Electronic Engineering, Duy Tan University, Da Nang 550000, Vietnam

**Keywords:** ANOVA, geometrical optimization, nanofluid, non-dominated sorting genetic algorithm, response surface methodology

## Abstract

Nanofluids in minichannels with various configurations are applied as cooling and heating fluids. Therefore, it is essential to have an optimal design of minichannels. For this purpose, a square channel with a cylinder in the center connected to wavy fins at various concentrations of an Al_2_O_3_ nanofluid is simulated using the finite volume method (FVM). Moreover, central composite design (CCD) is used as a method of design of experiment (DOE) to study the effects of three input variables, namely the cylinder diameter, channel width, and fin radius on the convective heat transfer and pumping power. The impacts of the linear term, together with those of the square and interactive on the response variables are determined using Pareto and main effects plots by an ANOVA. The non-dominated sorting genetic algorithm-II (NSGA-II), along with the response surface methodology (RSM) is applied to achieve the optimal configuration and nanofluid concentration. The results indicate that the effect of the channel width and cylinder diameter enhances about 21% and 18% by increasing the concentration from 0% to 5%. On the other hand, the pumping power response is not sensitive to the nanofluid concentration. Besides, the channel width has the highest and lowest effect on the heat transfer coefficient (HTC) and pumping power, respectively. The optimization for a concentration of 3% indicates that in *Re* = 500 when the geometry is optimized, the HTC enhances by almost 9%, while the pumping power increases by about 18%. In contrast, by increasing the concentration from 1% to 3%, merely an 8% enhancement in HTC is obtained, while the pumping power intensifies around 60%.

## 1. Introduction

Nanofluids are nanoparticle suspensions dispersed in a host fluid, usually including water, oil, and ethylene glycol. Nanofluids, which are extremely useful with remarkable thermal properties, are innovative fluids with various applications in the industry. Hence, it has been exciting for many scholars [1,2]. Several studies have been conducted, and it is still undergoing at universities and research centers in this field. Additionally, many scientists have also reviewed and classified the studies performed in this area [3,4]. In recent years, minichannels and microchannels have attracted much attention, and scholars have investigated the combination of different configurations using nanofluids as cooling or heating fluids [5,6]. A summary of the studies on the employment of nanofluids in various shapes is presented in Table 1.

Furthermore, research has been conducted to predict [7,8,9] and optimize [10,11,12] the desired performance of nanofluids in multiple conditions. It should be noted that a few studies have been conducted on the optimization of configuration to obtain the conditions with the expected thermohydraulic attributes. Nanofluids promise thermal fluids. However, heat transfer can be intensified by employing nanofluids and using an optimized configuration simultaneously.

In the case of single-objective problems, the main target of solving problems is to improve the index of single performance so that either the maximum or the minimum value utterly indicates the outcome quality [13]. Nevertheless, in several conditions, relying on merely one index in an optimization problem to evaluate a hypothetical response would not be possible. Therefore, there is a need for defining more than one objective function or index to then optimize all of them simultaneously. The non-dominated sorting genetic algorithm-II (NSGA-II) is a standard algorithm used in intelligence optimizations. The suggested method by Box and Wilson, namely the response surface methodology (RSM), is a mathematical and statistical approach that can be employed for studying the effects of diverse input variables at various levels.

Moreover, RSM utilizes a design such as a central composite design (CCD) to fit a model [14]. The efficiency of the model could be verified by checking the tools provided by an analysis of variance (ANOVA). The Pareto and main effects plots could be employed to assess the impact of the input variables on the response variables.

Many studies performed RSM to model the thermophysical properties of nanofluids [22,23,24]. However, a limited number of them applied optimization in configuration while employing a nanofluid as a working fluid [25,26,27]. Kumar and Dinesha [28] optimized the thermal characteristics of heat transfer improvement in a double pipe heat exchanger using RSM. Rashidi et al. [29] optimized the flow of a nanofluid around an equilateral triangular obstacle in which the optimum conditions for the maximum heat transfer rate and the minimum drag coefficient were predicted using RSM. The multi-objective optimization of the corrugated tube with a loose-fit twisted tape through RSM is done to correlate the Reynold number, twisted ratio, and clearance ratio with the Nusselt number ratio, friction factor ratio, and overall heat transfer performance to estimate the optimum design range of a heat exchanger [30,31]. The optimization of the viscosity and thermal conductivity of the Al_2_O_3_/water, CuO/water, SiO_2_/water, and ZnO/water nanofluids is performed using RSM and NSGA-II, in which the thermal conductivities of the Al_2_O_3/_water and CuO/water nanofluids determine the maximum increment at different temperatures and volume fractions [32]. It also should be noted that the current optimization method can be used for entropy generation [33].

According to the review of the relevant literature, it is concluded that using a nanofluid enhances the HTC [34,35,36]. In contrast, nanofluids not only improve the convective heat transfer but also intensify the pressure loss and, consequently, the pumping power [37,38]. Several studies have been conducted on employing nanofluids in various configurations [39,40,41]. However, a few studies are carried out to employ the nanofluid in an optimized configuration. The objective of this research is to perform a method for multi-objective optimization of configuration of a minichannel while having the Al_2_O_3_/water nanofluid using the NSGA-II algorithm. This nanofluid is widely employed in this area and has many applications. The mathematical models for predicting the maximum HTC and minimum pumping power of the nanofluid are presented using an ANOVA. 

## 2. Material and Methods

**Figure 1:**
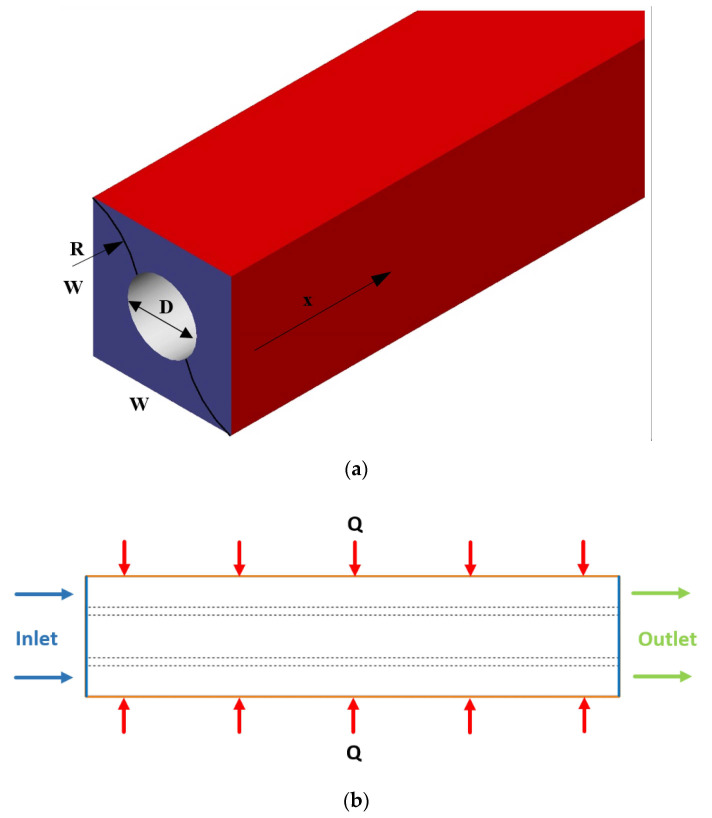
The study case: (**a**) isometric view of the channel with an inner cylinder and with a wavy fin; (**b**) side view.

The present numerical research is to study the heat transfer and pressure drop of the water and nanofluid flow containing Al_2_O_3_ nanoparticles in a square channel with a cylinder in the center, consisting of a fin with a constant heat flux condition (see Figure 1a). To simulate the laminar flow regime in a constant mass flow rate of 0.03 Kg/s, the finite volume method (FVM) is applied. Furthermore, the constant uniform heat flux on the channel wall is considered as a thermal boundary condition (see Figure 1b). A numerical investigation is carried out for the nanofluid with concentrations of 0% to 5% through a straight channel. Also, the basic dimension of minichannel is tabulated in Table 2. 

### 2.1. Multi-Objective Optimization

#### 2.1.1. Design of Experiments

To reduce the number of tests, time, and cost, the design of experiments (DOE) is an essential tool to solve multi-variable engineering problems. Further, CCD is a second-order design in which merely three levels of every variable are needed. CCD provides a survey on the effects of each variable and their interaction throughout the responses by means of fewer experimental runs compared with a full-factorial model. Additionally, CCD can be used to predict and optimize responses [42]. In the current study, the CCD consists of 15 numerical experiments at three independent input variables, including cylinder diameter, channel width, and fin radius with three levels. The design space for each input variable is based on the design limitations; therefore, the possible range is selected. Table 3 demonstrates the actual values of the three independent variables. The experimental layout, which is implemented in the current study in actual form, is tabulated in Table 4.

#### 2.1.2. Response Surface Methodology

**Figure 2:**
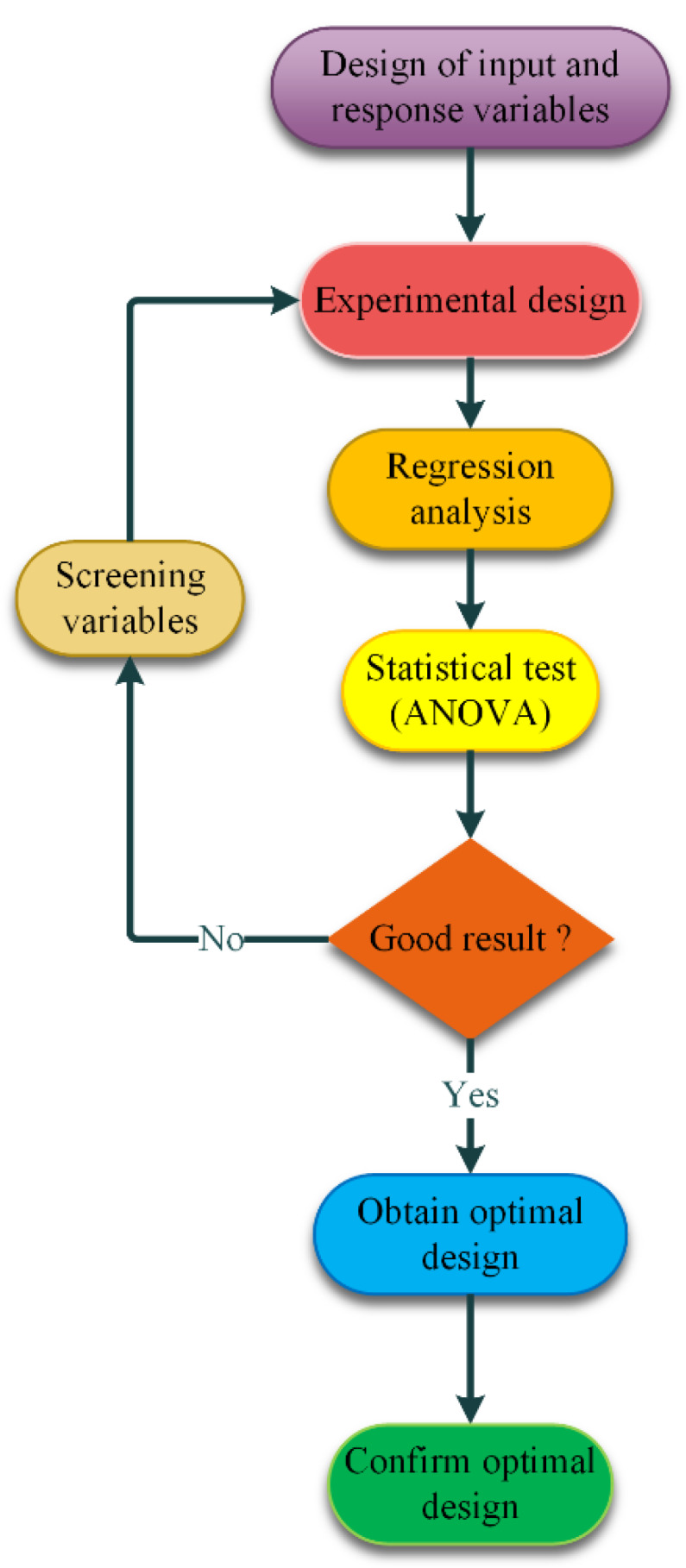
The response surface methodology (RSM) flowchart.

The RSM is widely adopted as a statistical approach applied for experimental purposes. The RSM performs by conducting a statistical design of the experiments, followed by assessing the coefficients in the mathematical model, and by predicting the responses and sufficiency examining of the model. In the RSM, the quantitative interaction pattern response variables and independent variables can be equally interpreted. The flowchart of the RSM is demonstrated in Figure 2.

A second-order polynomial equation is assigned to the numerical results that in its general form is

(1) $$Y=\beta_{0}+\sum_{i=1}^{k}\beta_{i}X_{i}+\sum_{i=1}^{k}\beta_{ii}X_{i}^{2}+\sum_{i=1}^{k}\sum_{i\neq j=1}^{k}\beta_{ij}X_{i}X_{j}+\varepsilon$$

where *Y* assigns a response, *X_i_* and *X_j_* are the independent variables, *β*_0_ is the constant coefficient, the coefficient of the linear term together with those of the quadratic and the interaction are considered as *β_i_*, *β_ii_*, and *β_ij_*, respectively, *k* denotes the number of independent variables that in this study are equal to 3, and *ɛ* is used for error [43].

#### 2.1.3. NSGA-II Algorithm

The NSGA-II is known as the secondary type of NSGA algorithm. Both types of NSGA are generated based on the genetic algorithm and extracted from the evolution theory of Darwin as well as genetic science. These two algorithms utilize the rule of “survival of the fittest” on responses to problems, intending to achieve proper outcomes. In the initial type of the algorithm, the users are supposed to find the suitable quantity through changing the σ shared in different problems. However, to avoid any accumulation of population members and support of every level of design interval, the subprogram of the crowding distance (CD) is used in the second version of NSGA.

Through the optimization with two objective functions, the allocated value of the CD to each chromosome is precisely equal to the perimeter of a rectangular created by the next and previous chromosomes. The magnitude of the CD of each population member, in comparison with the next and former members, together with the first and last population members, is calculated based on Equation (2) [7].

(2) $$d_{ij}=\frac{\left|f_{1}^{k}-f_{1}^{j}\right|}{f_{1}^{max}-f_{1}^{min}},\;d_{ik}=\frac{\left|f_{2}^{k}-f_{2}^{j}\right|}{f_{2}^{max}-f_{2}^{min}},\;CD_{i}=d_{ij}+d_{ik}$$

The process of optimization is in a way that the N numbers of the primary populations are firstly generated by random. Then, the values of the objective functions are calculated for the primary population, and these members are classified, and their CD is specified. Parents are selected based on their rank and CD scale through the binary tournament selecting method, crossover, and mutation operators that are used on them. This new population is combined with the prior population, and the sorting operation is repeated. Between the total available populations, which their members are even more than the primary population, the N numbers of the population’s upper members are chosen for the other generation. Upper fronts are initially determined to select the population of the other generation. Then, if by selecting another front, the number of population members gets more than the N, an adequate number of that front is selected based on the CD scale.

**Figure 3:**
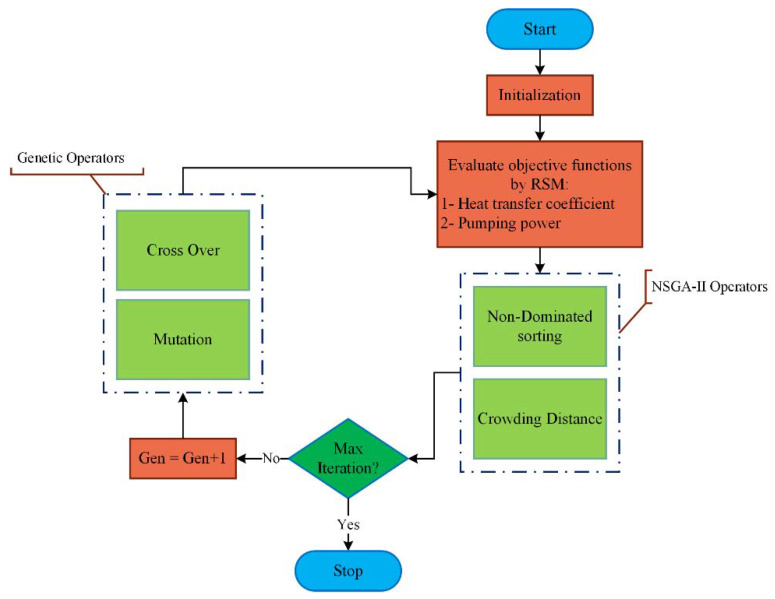
Flowchart of the non-dominated sorting genetic algorithm-II (NSGA-II).

The flowchart of NSGA-II is illustrated in Figure 3. The optimization method utilizes the RSM to assign the function of fitness, as shown in Figure 3. Besides, new operators, namely, mutation and crossover, are applied for the populations generating. By the end, the process of optimization would be finalized with the condition of some iterations.

### 2.2. Governing Equations

For modeling the laminar flow, the Navier–Stokes equations are employed to solve the performance of the fluid. As a result, the governing equations at the steady-state flow are as below [44]:

Mass conservation:

(3) $$\nabla \cdot (\rho_{nf}V_{nf})=0$$

Momentum conservation:

(4) $$\nabla \cdot (\rho_{nf}VV_{nf}V_{nf})=-\nabla P_{nf}+\nabla \cdot (\mu_{nf}\nabla V_{nf})$$

Energy conservation:

(5) $$\nabla \cdot (\rho_{nf}V_{nf}C_{p,nf}T_{nf})=\nabla \cdot (k_{nf}\nabla T_{nf})$$

where *ρ* (kg m^−3^) and *k* (W m^−1^ K^−1^) represent the density and thermal conductivity, respectively. *μ* (N m^−2^ s) and *C_p_* (J kg^−1^ K^−1^) show the dynamic viscosity and the specific heat, respectively. Meanwhile, *V* (m s^−1^), *T* (K), and *P* (N m^−2^) are the velocity, temperature, and pressure, respectively.

### 2.3. Nanofluid Properties

The base fluid is water with temperature-dependent properties. Its viscosity is calculated via Equation (6) [45]. Further, Equation (7) calculates the thermal conduction of water, while its density and specific heat are evaluated by Equations (8) and (9), respectively [1].

(6) $$\mu_{bf}=0.00002414\times 10^{\left(\frac{247.8}{\left(T-140\right)}\right)}$$

(7) $$k_{bf}=2.33417-0.0328575T+0.000185702T^{2}$$

(8) $$\rho_{bf}=753.2+1.88T-3.570\times 10^{-3}T^{2}$$

(9) $$c_{p,bf}=-29664.8+403.224T-1.78895T^{2}+0.00349982T^{3}-0.00000254434T^{4}$$

To assess the density and specific heat of the nanofluid, Equations (10) and (11) are used, respectively [1].

(10) $$\rho_{nf}=(1-\varphi )\rho_{bf}+\varphi \rho_{p}$$

(11) $$c_{p,nf}=\frac{\varphi \rho_{p}c_{p,p}+(1-\varphi )\rho_{bf}c_{p,bf}}{\rho_{nf}}$$

The nanofluid viscosity is attained by employing the below model [46]:

(12) $$\frac{\mu_{nf}}{\mu_{bf}}=1+1631{\left(\frac{\varphi }{1-\varphi }\right)}^{2.8}$$

Vajha and Das [47] offered Equation (13) to evaluate the thermal conductivity of the nanofluid. This model consists of two terms, which are demonstrated in Equations (14) and (15). The first and second terms, respectively, denote the static and dynamic part of the model. Equation (15) is for the Brownian motion of the nanoparticles.

(13) $$k_{nf}=\frac{k_{p}+2k_{bf}-2\left(k_{bf}-k_{p}\right)\varphi }{k_{p}+2k_{bf}+\left(k_{bf}-k_{p}\right)\varphi }k_{bf}+5\times 10^{4}\beta \varphi \rho_{bf}c_{pbf}\sqrt{\frac{kT}{\rho_{b}d_{p}}}f\left(T,\varphi \right)$$

(14) $$f(T,\varphi )=(2.8217\times 10^{-2}\varphi +3.917\times 10^{-3})(\frac{T}{T_{0}})+(-3.0669\times 10^{-2}\varphi +3.91123\times 10^{-3})$$

(15) $$\beta =8.4407{\left(100\varphi \right)}^{\left(-1.07304\right)};\;1\%\leq \varphi \leq 100\%,\;298K\leq T\leq 363K$$

*T*_0_ is equal to 273 K and indicates the reference temperature [48].

The properties of the aluminum oxide particles are presented in Table 5 [48].

### 2.4. Boundary Conditions

A laminar and steady-state flow of the nanofluid with a uniform velocity and temperature profile with different concentrations (the inlet temperature is 25 °C) is considered. A constant and uniform heat flux, as much as 200 W/m^2^ is applied as the thermal condition on the channel walls. Furthermore, the relative pressure is set to zero at the outlets of the channel (Figure 1b). Also, the no-slip condition is considered on the walls. The boundary conditions are described in mathematical forms as follows:

At the inlets: *T = T*_0_, *v = v*_0_;

At the outlets: *P_gage_* = 0;

At the walls: *v =* 0.

Equation (16) evaluates the Reynolds number as follows:

(16) $$Re=\frac{4m^{.}}{\pi D_{h}\mu }$$

where *m*· denotes the mass flow rate, and *D_h_* is the hydraulic diameter calculated by applying Equation (17).

(17) $$D_{h}=\frac{4W^{2}-\pi D^{2}}{4W+\pi D}$$

To evaluate the HTC, the Equation (16) is applied [48].

(18) $$h=\frac{q^{\prime \prime }}{T_{w}-T_{m}}\;\mathrm{and}\;T_{m}=\frac{T_{in}+T_{out}}{2}$$

The temperatures of the fluid are *T_in_* and *T_out_* in the outlet and inlet, respectively. Furthermore, *q*^”^ denotes the heat flux applied to the channel wall, and *T_w_* and *T_m_* indicate the average wall temperature and the average fluid temperature, respectively.

To evaluate the pumping power, Equation (19) is applied.

(19) P=QΔP

where *Q* represents the rate of fluid flow, and *ΔP* indicates the pressure loss.

### 2.5. Numerical Solution

The control volume method is used to solve the present case numerically. To couple the pressure and velocity, the SIMPLE algorithm is employed. Moreover, the second-order upwind scheme is utilized to solve the continuity, momentum, and energy equations. For all variables, the minimum divergence criteria are assumed to be 10^−5^.

### 2.6. Grid Generation and Grid Independency

**Figure 4:**
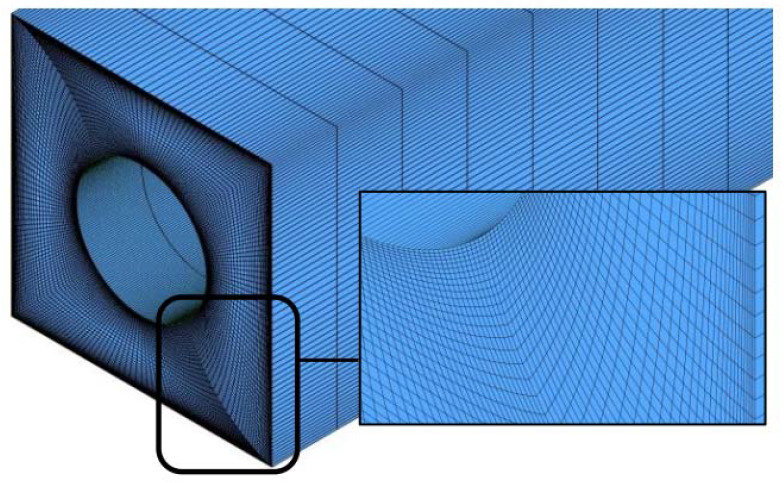
Channel gridding.

For evaluating the mesh independency, the effect of different grids on the HTC at the channel exit is assessed. According to Figure 4, finer cells along the radius to the walls are employed where gradients are high. Water enters the channel at a temperature of 25 °C and *Re* = 500, as shown in Figure 1, and the properties are calculated by Equations (6)–(9). A constant heat flux of 200 W/m^2^ is applied to the channel walls. The HTC at the channel exit is achieved by the different elements represented in Table 6. It is seen in Table 6 that minifying grids more than 2673173 have no noticeable effect on the HTC. Hence, this grid is adopted for further simulation in the current study. 

## 3. Results and Discussion

### 3.1. Validation

**Figure 5:**
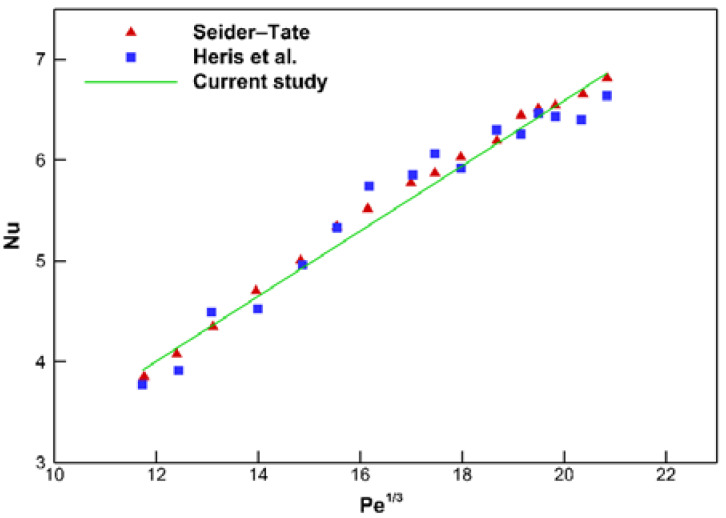
Comparison between the current simulation and those reported by Sieder–Tate [49] and Heris et al. [50] for water at the constant wall temperature.

For the validation of the current simulation, the numerical results are evaluated with the correlation presented by Sieder–Tate [49] and the experimental data from Reference [50] for laminar flow with the constant wall temperature boundary condition. Laminar water flows in a 1-m long tube with a 6 mm diameter under the constant wall temperature condition, and the achieved results are shown in Figure 5. According to Figure 5, the almost same response from the simulation of the data from the experimental and theoretical study [49,50] highlights the accuracy of the current simulation.

### 3.2. DOE Results

The HTC and pumping power are selected as the two response variables in every design of an experiment’s numerical simulation, including water-based and nanofluids in the concentration of 1%, 3%, and 5%. The magnitude of every response variable is tabulated in Table 7.

### 3.3. Analysis of Variance

**Figure 6:**
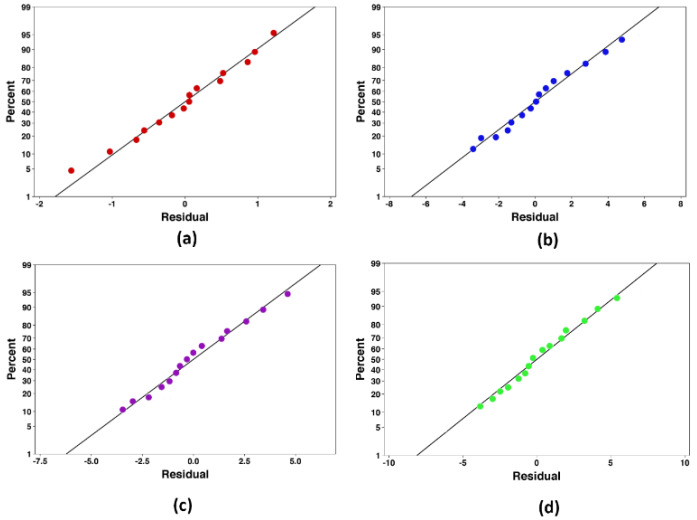
Normal probability plot of the residuals for the heat transfer coefficient (HTC), (**a**): *φ* = 0%, (**b**): *φ* = 1%, (**c**): *φ* = 3%, and (**d**): *φ* = 5%.

**Figure 7:**
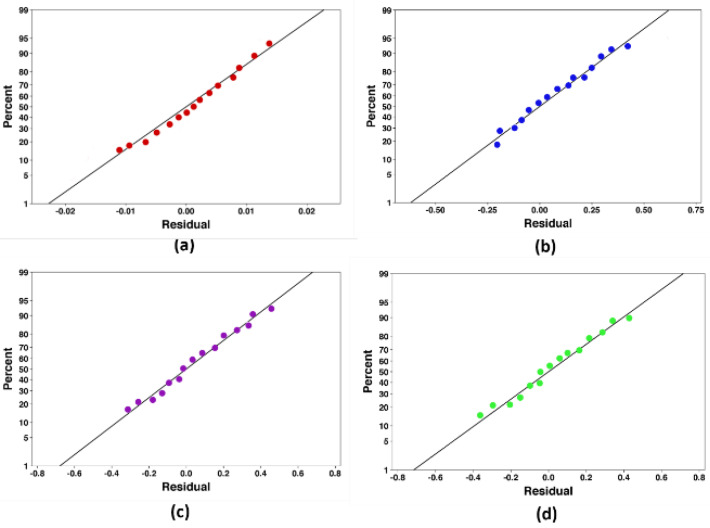
Normal probability plot of the residuals for the pumping power, (**a**): *φ* = 0%, (**b**): *φ* = 1%, (**c**): *φ* = 3%, and (**d**): *φ* = 5%.

An analysis of variance is employed to assess the data using the least square method. All required ANOVA assumptions consisting of case independence, normality of residuals distribution, and equality of variances are analyzed to determine the goodness of the fitted model. Therefore, the residual plots are drawn, as shown in Figure 6 and Figure 7. The regression coefficient of the linear term, in addition to those of the quadratic and interaction, existed in the fitted model, and their impacts are assessed. All terms of the fitted model are verified utilizing their probability magnitudes at a 95% confidence level (*p*-value < *0.05*). To acquire the best model, the adjusted coefficient of determination (*R*^2^*_adj_*) is applied to assess the efficiency of the models. Once the ultimate model is reached, the main effect plots are performed to illustrate the effects of the input variables on the responses. The value of *R*^2^*_adj_* for each response is tabulated in Table 8.

The second-order equations with all the mentioned terms are provided due to the results of the numerical experiments to create a mathematical model to predict the value of the heat transfer coefficient and pumping power for each studied condition. The relevant correlations are tabulated in Table 9.

**Figure 8:**
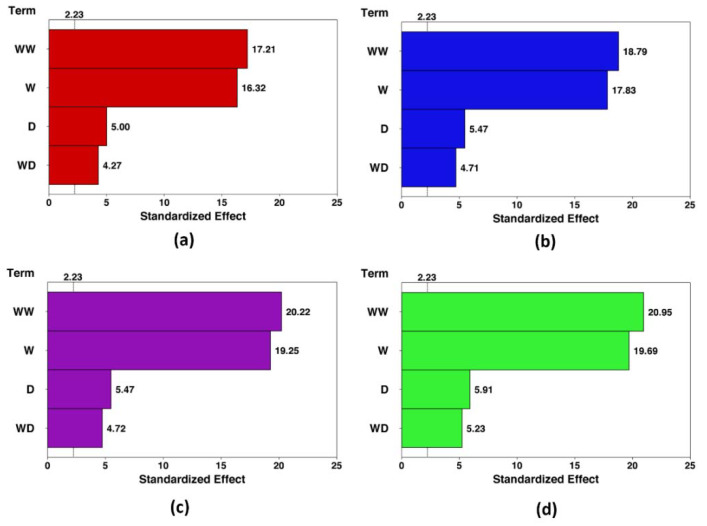
Pareto chart of the standardized effects (response is HTC, *α* = 0.05). (**a**): *φ* = 0%, (**b**): *φ* = 1%, (**c**): *φ* = 3%, and (**d**): *φ* = 5%.

**Figure 9:**
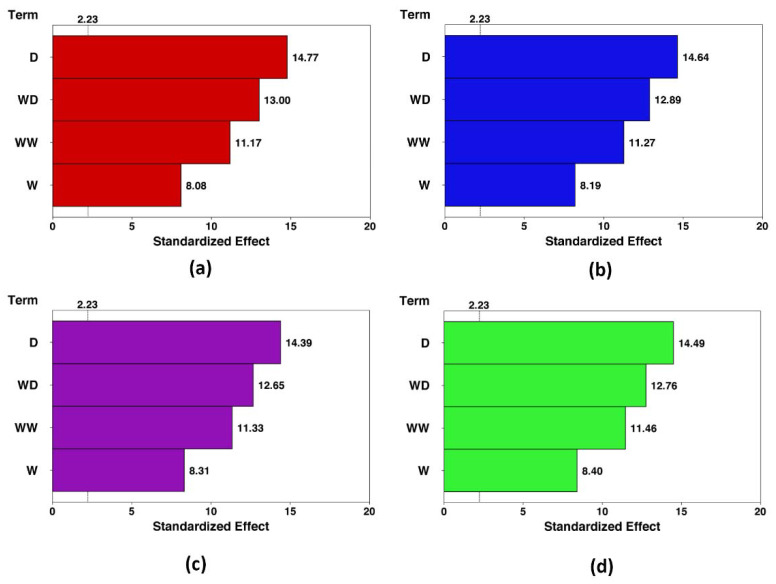
Pareto chart of the standardized effects (response is pumping power, *α* = 0.05). (**a**): *φ* = 0%, (**b**): *φ* = 1%, (**c**): *φ* = 3%, and (**d**): *φ* = 5%.

The Pareto charts are drawn to demonstrate the effect of each term on both responses. These charts for the HTC and pumping power in each condition are shown in Figure 8 and Figure 9, respectively. The Pareto charts show that the effect of the optimization is more intense in the higher concentration. The effect of the channel width and cylinder diameter increase around 20.8% and 18% by enhancing the concentration from 0% to 5%. In contrast, the pumping power response is not sensitive to the nanofluid concentration. Further, the channel width has the highest and lowest impact on the HTC and pumping power, respectively.

**Figure 10:**
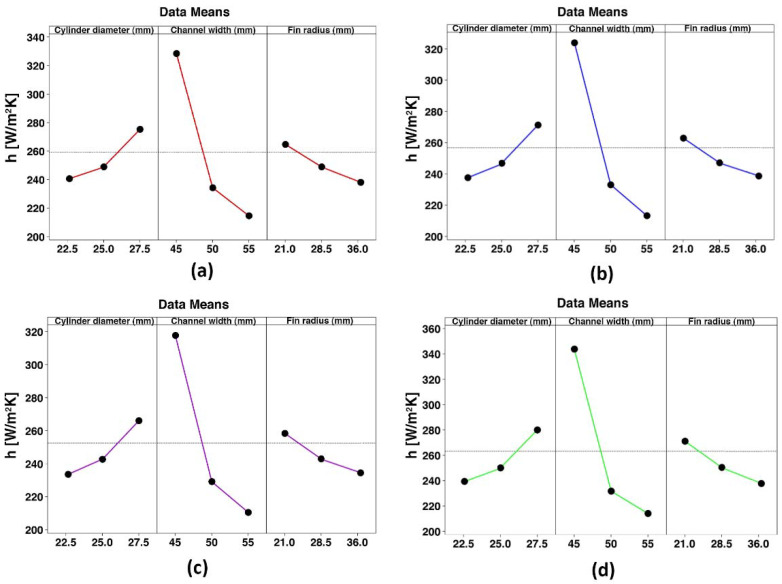
Main effects plots for the HTC. (**a**): *φ* = 0%, (**b**): *φ* = 1%, (**c**): *φ* = 3%, and (**d**): *φ* = 5%.

**Figure 11:**
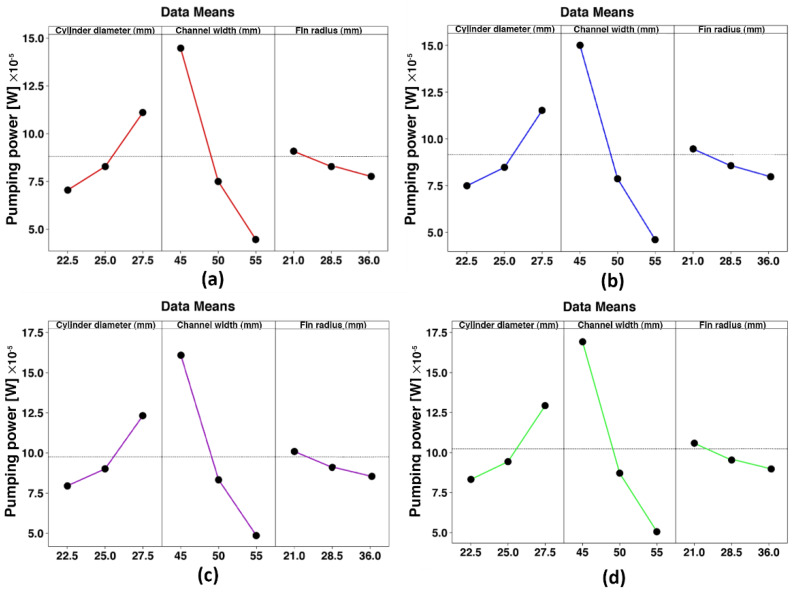
Main effects plots for the pumping power. (**a**): *φ* = 0%, (**b**): *φ* = 1%, (**c**): *φ* = 3%, (**d**): *φ* = 5%.

The main effects of the plots are drawn to better identify the impact of the input variables on the responses, as shown in Figure 10 and Figure 11. As can be seen, the fin radius has the least effect on the HTC and pumping power compared with the channel width and cylinder diameter. Moreover, it should be noted that a wavy fin with a smaller radius will result in better thermal performance because the area of heat transfer is increased. The channel side is the most effective factor in all concentrations. Figure 10 illustrates the HTC in terms of the channel dimension change, where it denotes that at *φ* = 5%, by decreasing the W from 50 to 45 mm, the HTC improves by about 49%. Further, the cylinder diameter change indicates the enhancement of about 12% by increasing the D from 25 to 27.5 mm. Regarding Figure 11, decreasing the W from 50 to 45 mm intensifies the pumping power by about 94%. In comparison, it increases by about 37% with the increment of the D from 25 to 27 mm. All in all, for the enhancement of the same magnitude in the HTC, the W causes a smaller increase in the pumping power. Hence, in the Pareto chart, the W has the highest and lowest effect on the HTC and the pumping power, respectively. Figure 10 and Figure 11 show the result that an increment of the D accompanied by the W decrement can have, which results in a hydraulic diameter reduction. The mass flow rate increases the Re, and higher HTCs are expected in higher Reynolds numbers. 

As it is mentioned, the RSM can not only be utilized to illustrate the impact of some input variables on certain response variables but is also an easy way to predict the optimal values. In this study, the optimization is performed to achieve the optimum geometry by the NSGA-II method based on the controlled elitism concepts. The pros of the NSGA-II overweighting method are a regularly distributed Pareto-optimal front and suitable for detecting. The Pareto-optimal front is used for non-convex multi-objective problems to avoid time-consuming delays. Moreover, it is applied to represent the Pareto-optimal solutions in a single run. This method also supports multiple objectives and constraints and targets at reaching the global optimum (Figure 3). In this method, after generating 600 samples per iteration, three candidate points are predicted, while a maximum of 20 iterations is set up [51]. The objective functions in the current survey are selected to maximize the HTC and minimize the pumping power with the same importance level. Table 10 represents the candidate points generated by the RSM. 

**Figure 12:**
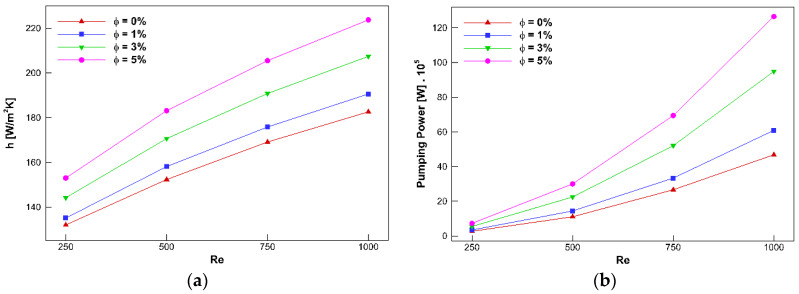
Convective HTC (**a**) and pumping power (**b**) versus Reynolds number for concentrations from 0% to 5% for the channel with a *H* = 45 mm and *D* = 22.5 mm.

According to Table 10, the optimum points to maximize HTC while minimizing the pumping power are determined. Adding the nanoparticles at a constant mass flow rate changes the Reynolds number. Thus, it would be better to assess the hydrothermal characteristics of the channel at constant Reynolds numbers instead of constant mass flow rates. Figure 12a demonstrates the convective HTC at the channel exit in terms of the Reynolds number at different concentrations for the channel with a *W* = 45 mm and *D* = 22.5 mm. It can be perceived that the convective HTC improves when the Reynolds number is enhanced.

Consequently, the convective HTC intensifies by almost 21.5% when the Reynolds number is increased from 250 to 1,000 at *φ* = 3%. Additionally, enhancing the concentration augments this parameter because adding the nanoparticles intensifies the thermal conductivity, which improves the rate of heat transfer. Therefore, when the concentration increases from 0% to 5% at *Re* = 1000, the convective HTC improves by about 46.2%.

Hydraulic characteristics, including the pumping power and pressure drop of the channel, also should be noted since they indicate the energy consumption rate needed for the operation of the channel. The pressure loss and heat transfer are enhanced by adding the nanoparticles; however, the pressure loss increment is undesirable.

Figure 12b shows the concentration effect on the pumping power in the different Reynolds numbers for the channel with a *W* = 45 mm and *D* = 22.5 mm. From the figure, it is expected to have a higher HTC while obtaining a smaller pumping power. It can be noted that the pumping power is intensified when either the Reynolds number or the concentration is increased. The velocity gradient increases when the Reynolds number enhances; therefore, the pressure loss augments. Moreover, increasing the Reynolds number enhances the velocity and leads to a higher rate of fluid flow. Consequently, the pumping power is signified by the Reynolds number (see Equation (19)). Therefore, the pumping power value at *Re* = 1000 is almost 17 times higher than that at *Re* = 250 for *φ* = 5%. Moreover, the viscosity, augmented by adding nanoparticles, increases the pressure loss, and as a result, the pumping power is raised. For instance, a 170% enhancement is seen in the pumping power when the concentration is increased from 0% to 5% at *Re* = 1000. Further, it should be noted that the impact of nanoparticle dispersion in the pumping power is more intense at higher Reynolds numbers.

**Figure 13:**
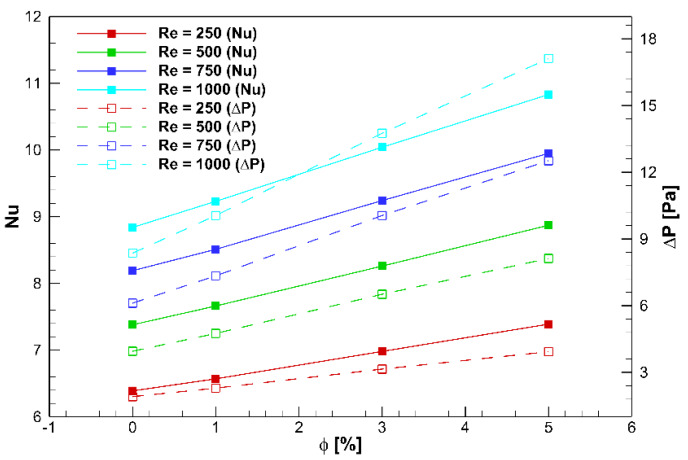
Variation of Nusselt number and pressure drop in terms of volume concentration at four Reynolds number obtained from the current optimized dimensions (*H* = 45 mm and *D* = 22.5 mm).

Figure 13 displays the effect of the volume concentration on the Nusselt number of the nanofluid for four Reynolds numbers. Accordingly, the Nusselt number in the different Reynolds numbers is signified with any rise in the volume concentration. Enhancing the particle density increases the momentum and HTC. It should be noted that the nanofluid viscosity intensifies when the particle volume concentration augments. As a result, by the concentration increment, the nanofluid pressure loss increases compared with the base fluid. Figure 13 also illustrates the variations in the pressure loss for Al_2_O_3_/water in terms of the volume concentrations at four Reynolds number. Like the base fluid, increasing the Reynolds number at a constant concentration raises the nanofluid pressure loss. For instance, by changing the Reynolds number from 500 to 1000 at a 3% volume concentration, the pressure loss intensifies by almost 70%. The slope of pressure loss experiences growth with the volume concentration.

**Figure 14:**
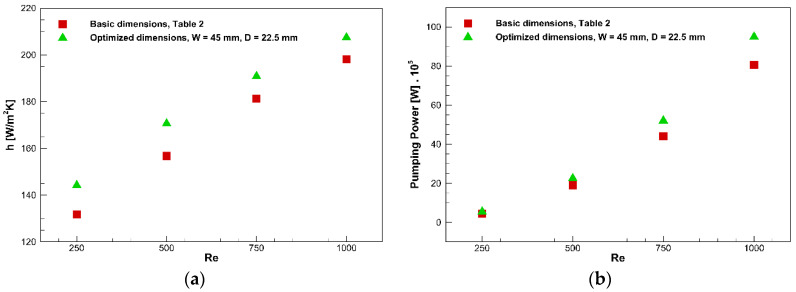
Comparison of the convection HTC (**a**) and pumping power (**b**) obtained from the current optimization and basic dimensions (Table 2) for the water/Al_2_O_3_ nanofluid at *φ* = 3%.

Figure 14 illustrates the convective HTC at the channel exit and the pumping power in terms of the Reynolds number for the channel with basic dimensions (Table 2) with a 3% concentration. The optimum values for the channel dimensions are obtained, and one of them is evaluated in Figure 14, such that the convective HTC enhances while the pumping power has a negligible increment. For instance, in Re = 500, when the geometry is optimized, the HTC enhances by almost 8.8%, while the pumping power rises by about 18%. In contrast, by increasing the concentration from 1% to 3%, only a 7.6% enhancement in HTC is achieved, while the pumping power rises by about 60%.

**Figure 15:**
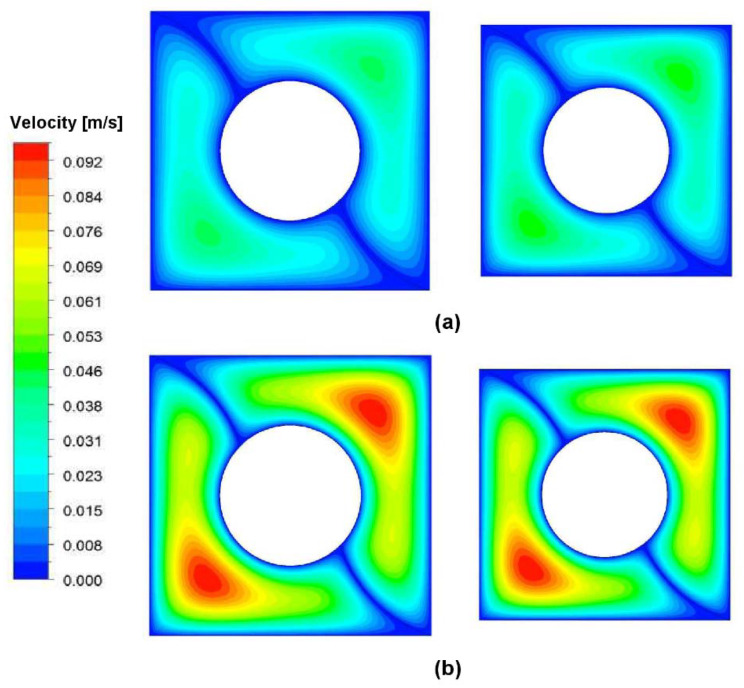
Velocity contour comparison between the channel with the base size (left Figure) and the optimum size (right Figure) at the channel exit at *φ* = 3% for: (**a**) *Re* = 500, and (**b**) *Re* = 1000.

For a better evaluation of the heat transfer variation with the geometry changing, the temperature contour at the channel exit is illustrated in Figure 15 for two Reynolds numbers at *φ* = 3%. It can be seen that the nanofluid experiences a lower temperature at the wall, for both Reynolds at an optimized channel. It shows a higher rate of heat transfer in the channel with the optimized dimensions. Moreover, the dimensions of the two channels are displayed in Table 11.

## 4. Conclusions

In the present research, the impacts of geometry properties of a minichannel including the cylinder diameter, channel width, and fin radius on the convective heat transfer coefficient and pumping power in concentrations of 0%, 1%, 3%, and 5% of Al_2_O_3_/water nanofluid is assessed. The second-order models are demonstrated to recognize the correlation between the independent and response variables. The results are presented based on the constant mass flow rate and a constant Reynolds number. The main results achieved from the current study are as follows:

- The *R*^2^*_adj_* is found to be more than 99% for each response in the various concentrations by the ANOVA;
- Applying the NSGA-II indicates that the effect of the channel width and cylinder diameter improves by about 20.8% and 18% through increasing the concentration from 0% to 5%;
- The pumping power response is not sensitive to the nanofluid concentration;
- The smaller the fin radius, the higher the HTC and pumping power;
- Channel width has the highest and lowest effect on the HTC and pumping power, respectively;
- The optimization for the concentration of 3% demonstrates that in Re = 500 when the geometry is optimized, the HTC enhances about 8.8%, while the pumping power increases by almost 18%. In contrast, by increasing the concentration from 1% to 3%, merely a 7.6% enhancement in the HTC occurs, while the pumping power intensifies around 60%.

## Figures and Tables

**Table 1:** Studies on employing nanofluids in different configurations.

Researchers	Nanofluid Type	Configuration Type	Main Results
Ahmadi et al. [5]	Water-Al_2_O_3_	Minichannel with cylinder, No fin, wavy fin, flat fin	Nanofluid concentration increment: thermal conductivity improvement. Heat transfer enhancement: 199.6%. Decreasing particles size: HTC increases
Bahiraei and Heshmatia [15]	Water-Ag	A liquid block heat sink	Using nanofluid at *φ* = 1% and *Re* = 500: temperature reduces by 2.21 °C with the least entropy of 56.2%. Increasing concentration from 0% to 1% at *Re* = 1500: convective HTC enhancement of 15.2%
Bahiraei et al. [16]	Water-CMC/TiO_2_	C shaped minichannel Straight minichannel	Concentration and *Re* increment: frictional entropy generation increases; thermal entropy generation decreases.
Liu et al. [17]	Water-Al_2_O_3_	curved duct	Al_2_O_3_ nanoparticle in a curved duct increase the convective heat transfer. Nanoparticle shape affects the convective heat transfer of nanofluid. Nanoplatelets show the highest convective heat transfer and pressure drop among all other shapes.
Bahiraei et al. [18]	hybrid nanofluid containing coated Fe_3_O_4_/CNT	Double-tube heat exchanger	Maximum heat transfer enhancement: 53.8%
Ghasemi et al. [19]	Water-Al_2_O_3_	minichannel heat sink	Nanofluid concentration increment: thermal resistance decreases. Thermal performance factor enhancement at *Re* 490 and *φ* = 1.5%: 1.24
Bahiraei and Majd [20]	Water-Al_2_O_3_	Triangular minichannel	*Re* increases from 100 to 500 at *φ* = 5%: HTC enhances by 56%. At *φ* = 5%, *Re* increment from 100 to 300: the thermal entropy generation rate decreases by 29.7%.
Bergman [21]	Water-Al_2_O_3_	minichannel heat sinks	Nanofluids are not useful in this application due to minimal enhancement.

**Table 2:** Description of the under-study channel, basic dimensions.

L (m)	W (mm)	D (mm)	R (mm)
3	50	50	25

**Table 3:** The design space for each input variable.

Input Variable	Symbol	Lower Bound	Basic Value	Upper Bound
Cylinder diameter (mm)	D	22.5	25	27.5
Channel width (mm)	W	45	50	55
Fin radius (mm)	R	21	28.5	36

**Table 4:** Experimental layout.

Expt. No.	Cylinder Diameter (mm)	Channel Width (mm)	Fin Radius (mm)
1	25	50	28.5
2	22.5	50	28.5
3	27.5	50	28.5
4	25	45	28.5
5	25	55	28.5
6	25	50	21
7	25	50	36
8	22.5	45	21
9	27.5	45	21
10	22.5	55	21
11	27.5	55	21
12	22.5	45	36
13	27.5	45	36
14	22.5	55	36
15	27.5	55	36

**Table 5:** Thermophysical properties of the Al_2_O_3_ nanoparticles [48].

Properties	*k* (W/mK)	*ρ* (kg/m^3^)	*Cp* (kJ/kgK)
Value	36	3600	765

**Table 6:** Cell number studied in mesh independent study.

Grid	Cell Number	HTC	Error
Grid 1	191185	116.979	-
Grid 2	1004566	116.198	−0.7%
Main Grid	2673173	115.962	−0.2%
Grid 4	4276219	115.961	0.0%

**Table 7:** Response values.

Expt. No.	*φ* = 0%		*φ* = 1%		*φ* = 3%		*φ* = 5%	
	h (W/m^2^K)	Pumping Power (×10^−5^) (W)	h (W/m^2^K)	Pumping Power (×10^−5^) (W)	h (W/m^2^K)	Pumping Power (×10^−5^) (W)	h (W/m^2^K)	Pumping Power (×10^−5^) (W)
1	234.25	7.51	233.25	7.77	229.48	8.23	231.08	8.61
2	230.88	6.26	229.26	6.47	225.67	6.84	228.58	7.12
3	238.96	9.17	237.25	9.52	232.90	10.11	235.671	10.58
4	326.87	13.88	322.41	14.51	316.59	15.56	342.95	16.36
5	214.40	4.37	213.19	4.52	209.85	4.77	213.48	4.98
6	234.75	7.51	233.09	7.78	229.06	8.24	231.79	8.61
7	233.91	7.51	232.32	7.78	228.23	8.24	231.04	8.61
8	315.36	11.08	311.46	11.56	306.31	12.36	329.19	12.97
9	348.94	17.88	343.25	18.72	335.09	20.10	365.94	21.17
10	208.90	3.79	208.00	3.91	206.16	4.10	209.72	4.26
11	220.94	5.14	218.75	5.33	215.41	5.66	218.62	5.89
12	306.55	11.09	303.94	11.56	299.16	12.37	319.85	12.98
13	344.78	17.88	339.14	18.72	331.36	20.10	361.57	21.17
14	208.18	3.80	207.80	3.92	205.83	4.09	209.75	4.26
15	220.48	5.13	218.00	5.32	215.29	5.65	218.33	5.88

**Table 8:** The percentage of the adjusted coefficient of determination for each response.

Concentration	HTC	Pumping Power
0%	99.44	99.60
1%	99.54	99.59
3%	99.59	99.58
5%	99.54	99.58

**Table 9:** Correlations of the heat transfer coefficient and pumping power.

Concentration	Heat Transfer Coefficient	Pumping Power
0%	h = 3805 − 147.48 W + 27.90 D + 1.4795 W^2^ − 0.475 WD	P = 83.0 − 5.523 W + 6.225 D +0.07260 W^2^ − 0.10915 WD
1%	h = 3672 − 141.83 W + 26.85 D + 1.4224 W^2^ − 0.4603 WD	P = 90.6 − 5.944 W + 6.546 D +0.07772 W^2^ − 0.11477 WD
3%	h = 3653 − 140.30 W + 24.61 D + 1.4014 W^2^ − 0.4227 WD	P = 102.5 − 6.607 W + 7.053 D +0.08572 W^2^ − 0.12357 WD
5%	h = 4747 − 186.98 W + 34.62 D + 1.8924 W^2^ − 0.610 WD	P = 109.4 − 7.046 W + 7.492 D +0.09143 W^2^ − 0.1314 WD

**Table 10:** Predicted candidate points provided by the response surface optimization.

	Candidate NO.	Cylinder Diameter (mm)	Channel Width (mm)	Fin Radius (mm)	h (W/m^2^K)	Pumping Power × 10^−5^ (W)
0%	1	22.50	45.00	21.00	314.16	11.1
	2	24.23	45.01	21.35	326.62	12.9
	3	22.58	45.39	24.52	298.43	10.7
1%	1	22.53	45.01	21.08	308.00	11.5
	2	22.71	45.10	23.04	306.10	11.6
	3	24.16	45.04	28.62	316.16	13.4
3%	1	22.50	45.00	21.00	307.85	12.4
	2	22.51	45.02	23.58	306.53	12.3
	3	22.70	45.00	26.04	307.48	12.6
5%	1	22.50	45.00	21.00	327.17	13.0
	2	22.53	45.01	23.79	326.03	12.3
	3	22.78	45.01	30.78	327.04	13.3

**Table 11:** The dimension of channels compared in Figure 15.

	L (m)	W (mm)	D (mm)	R (mm)
Base size	3	50	50	25
Optimum size	3	45	22.5	21

## Nomenclature

*Cp*	Specific heat, J/kg.K
*D*	Cylinder diameter, m
g	Gravity Acceleration, m/s^2^
*h*	Convective heat transfer coefficient, W/m^2^.K
k	Heat Conductivity, W/m.K
m	Mass, kg
*Nu*	Nusselt number
P	Pressure, Pa
*Q*	Heat flux, W/m^2^
*R*	Fin radios, m
Re	Reynolds Number
t	Time, s
*T*	Temperature, K
V	Velocity, m/s
*W*	Channel height, m
*x*	Axial length, m
Greek symbols	
ρ	Density, kg/m^3^
μ	Dynamic Viscosity, Pa.s
*φ*	Volume concentration
Subscripts	
***b*** *f*	Base fluid
*m*	mean
*nf*	nanofluid
P	Particle
Abbreviations	
*ANOVA*	Analysis of variance
*CCD*	Central composite design
*CD*	Crowding distance
*DOE*	Design of experiments
*FVM*	Finite volume method
*HTC*	Heat transfer coefficient
*NSGA*	Non-Dominated Sorting Genetic Algorithm
*RSM*	Response surface methodology
